# Cyanophage Engineering for Algal Blooms Control

**DOI:** 10.3390/v16111745

**Published:** 2024-11-06

**Authors:** Yujing Guo, Xiaoxiao Dong, Huiying Li, Yigang Tong, Zihe Liu, Jin Jin

**Affiliations:** 1 Beijing Advanced Innovation Center for Soft Matter Science and Engineering, Beijing University of Chemical Technology, Beijing 100029, China; 2 College of Life Science and Technology, Beijing University of Chemical Technology, Beijing 100029, China; 3 State Key Laboratory of Chemical Resource Engineering, College of Life Science and Technology, Beijing University of Chemical Technology, Beijing 100029, China

**Keywords:** cyanophage, cyanobacteria, cyanobacteria harmful algal blooms, cyanobacteria bloom management

## Abstract

Cyanobacteria represent a prevalent category of photosynthetic autotrophs capable of generating deleterious algal blooms, commonly known as cyanobacteria harmful algal blooms (cyanoHABs). These blooms often produce cyanotoxins, which pose risks to public health and ecosystems by contaminating surface waters and drinking water sources. Traditional treatment methods have limited effectiveness. Therefore, there is an urgent need for a new approach to effectively manage cyanoHABs. One promising approach is the use of cyanophages, which are viruses that specifically target cyanobacteria. Cyanophages serve as an effective biological control method for reducing cyanoHABs in aquatic systems. By engineering cyanophages, it is possible to develop a highly specific control strategy that minimally impacts non-target species and their propagation in the environment. This review explores the potential application of cyanophages as a strategy for controlling cyanoHABs. It includes the identification and isolation of broad-spectrum and novel cyanophages, with a specific focus on freshwater Microcystis cyanophages, highlighting their broad spectrum and high efficiency. Additionally, recent advancements in cyanophage engineering are discussed, including genome modification, functional gene identification, and the construction of artificial cyanophages. Furthermore, the current state of application is addressed. Cyanophage is a promising control strategy for effectively managing cyanoHABs in aquatic environments.

## 1. Introduction

In recent years, inadequate treatment of industrial wastewater, coupled with the impacts of global warming, has resulted in increased eutrophication of aquatic ecosystems. As a result, cyanobacteria have grown excessively, causing outbreaks of cyanobacteria harmful algal blooms (cyanoHABs) across the globe [1,2]. The prevalence of cyanoHABs is escalating worldwide, resulting in various adverse impacts on local ecosystems and water resources [2,3].

**Figure 1:**
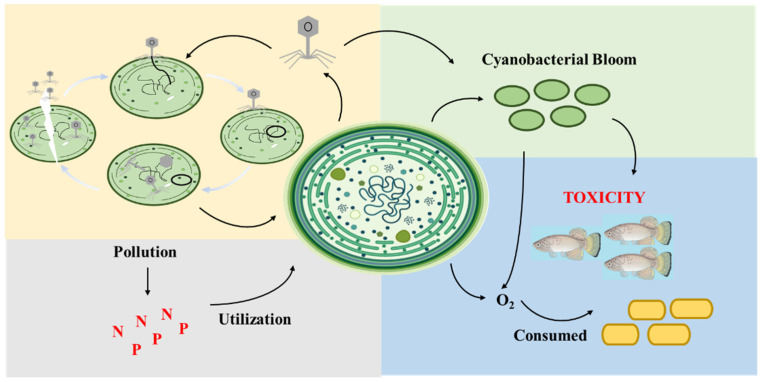
Cyanobacteria bloom and ecological cycle of cyanobacteria.

Cyanobacteria, a group of photosynthetic autotrophs, are widely distributed across various environments and are considered one of the most ubiquitous organisms on Earth [1]. Notably, as illustrated in Figure 1, cyanobacterial blooms, particularly those induced by *Microcystis*, present a significant concern [1,4]. These blooms deplete water of dissolved oxygen and lead to elevated levels of cyanobacterial toxins, including hepatotoxins and neurotoxins [3,5]. These toxins exhibit immunotoxic, teratogenic, carcinogenic, and mutagenic properties, resulting in a broad spectrum of acute and chronic health effects [6,7,8]. For instance, a high incidence of primary liver cancer (PLC) has been linked to the presence of cyanobacterial blooms in drinking water reservoirs [9]. Furthermore, the implications of cyanobacterial toxins have been extensively documented in numerous cases in both the United States and Australia [4]. Similarly, in China, several critical freshwater lakes, such as Lake Taihu, Lake Chaohu, and Lake Dianchi, are experiencing severe pollution issues [4].

Currently, the primary treatment method for controlling and mitigating cyanoHABs is to restrict nutrient loading into the aquatic systems [1,10]. In addition to traditional salvage methods, chemical approaches include the use of chemical agents [11] and ferrate (IV) oxidation [12]. However, these methods introduce additional chemicals into the water, which can harm the aquatic environment and jeopardize the health of both animals and plants. Physical methods for controlling algal blooms include aeration [13] and ultrasonic treatment [14], but these approaches can be costly and inefficient. Conventional biological methods involve cultivating aquatic plants to effectively absorb excess nitrogen and phosphorus. Additionally, these methods utilize antagonistic interactions to promote competitive growth inhibition, employ filter feeders for algal removal [15], and incorporate materials [16]. However, the growth rate of aquatic plants is often too slow to effectively eliminate cyanobacteria, and filter-feeding animals face challenges in consuming cyanobacteria due to their toxic effects. This toxicity complicates the use of antagonistic methods for inhibition. In summary, while various methods exist for controlling algal blooms, each approach has inherent limitations, making the comprehensive resolution of this issue a significant challenge.

Cyanophages are viruses that specifically target cyanobacteria [3,17]. Their ability to infect multiple genera of cyanobacteria presents new opportunities for managing cyanoHABs. Unlike traditional methods, cyanophages are cost-effective, easily accessible, simple to cultivate, and have a reduced tendency to cause additional environmental disturbances or disrupt ecological balance. With advancements in synthetic biology, there is significant potential to isolate and discover novel and diverse cyanophages, apply engineering principles to rationally design and modify these viruses, and comprehensively utilize artificial cyanophages for bloom control. This review focuses on the isolation of novel cyanophages, advancements in the genetic modification of cyanophages, and their practical applications, aiming to provide a new research pathway for effective bloom management.

## 2. Mining Broad-Spectrum and Efficient Cyanophages

Cyanophages are classified into three families: *Myoviridae*, *Siphoviridae*, and *Podoviridae*. Cyanobacterial blooms typically occur due to the proliferation of multiple dominant cyanobacterial species. Consequently, obtaining a diverse range of cyanobacteria is crucial for managing these blooms. While cyanophages exhibit host specificity, a few can infect cyanobacteria across different genera [18]. However, these instances are less common in freshwater environments. Our primary focus is on the potent infection capabilities and broad-spectrum infectivity of cyanophages in freshwater ecosystems.

*Microcystis* is a major harmful cyanobacterium that often causes large-scale cyanobacterial bloom outbreaks [19]. The most prevalent dominant algal species in these aquatic blooms are *M. wesenbergii*, *M. flos-aquae*, and *M. aeruginosa* [20]. Currently, the development of isolation and identification procedures for freshwater cyanophages is moving relatively slowly. According to the GenBank database, approximately 250 cyanophage genomes have been published globally, with the majority of the hosts being marine *Prochlorococcus* and *Synechococcus* cyanophages [21]. Prochlorococcus and Synechococcus cyanophages. In contrast, only about 40 freshwater cyanophages have been reported, sequenced, and annotated [22], of which only 13 are associated with Microcystis (see Table 1). These include ΦMHI42 [18], MaMV-DC [23], Ma-LMM01 [24], Mic1 [25], Ma-LBP [26], PhiMa05 [27], vB_MweS-yong2 [20], Me-ZS1 [28], MinS1 [29], Mae-Yong924-1 [30], Mae-Yong1326-1 [20], YongM [31], and Mwe-Yong1112-1 [32]. However, ΦMHI42 and Ma-LBP have not been sequenced, and only eight strains are classified as broad-spectrum cyanophages, which include ΦMHI42, MaMV-DC, Me-ZS1, MinS1, Mae-Yong924-1, Mae-Yong1326-1, YongM, and Mwe-Yong1112-1. These cyanophages can lyse multiple cyanobacterial strains (see Table 1). Mwe-Yong1112-1 was able to lyse 23 cyanobacterial strains from four different orders: *Chroococcales*, *Nostocales*, *Oscillatoriales*, and *Synechococcales* [32]. MinS1 could lyse 19 strains across *Chroococcales*, *Nostocales*, *Oscillatoriales*, *Hormogonales*, and *Synechococcales* [29]. YongM was effective against 18 cyanobacterial strains from four orders: *Chroococcales*, *Nostocales*, *Oscillatoriales*, and *Synechococcales* [31]. Me-ZS1 could lyse 12 strains from the taxonomic orders *Chroococcales, Nostocales*, and *Oscillatoriales* [28]. Mae-Yong1326-1 was capable of lysing seven cyanobacterial strains from three orders: *Chroococcales*, *Nostocales*, and *Oscillatoriales* [20]. Mae-Yong924-1 could lyse six diverse cyanobacterial strains from three orders: *Chroococcales*, *Nostocales*, and *Oscillatoriales* [30]. ΦMHI42 was able to lyse *Microcystis* and *Planktothrix* [18]. It has been reported that MaMV-DC infects and lyses *M. aeruginosa* FACHB-524, *M. flos-aquae* TF09, *M. aeruginosa* TA09, and *M. wesenbergii* DW09. Furthermore, MaMV-DC is genus-specific rather than strain-specific [33].

Cyanophages often take 4 to 16 days to completely eliminate their host bacteria, according to previous studies [18,27,34]. However, some effective cyanophages have already been isolated, such as Lbo240-yong1, which caused the cultures of its host, *L. boryana* FACHB-240, to become fully yellow within 1 day of infection. Lbo240-yong1 is classified as a freshwater cyanophage, not a *Microcystis* cyanophage [35]. As shown in Table 1, Mwe-Yong1112-1 completely lysed 7 of the 23 host algae within 3 days [32]. The host culture infected by YongM turned yellow in only 8 h [31]. Meanwhile, the one-step growth curve is an essential parameter for determining cyanophage infection efficiency. To date, only seven strains of *Microcystis* phage have been reported (Table 1). The latent period of MinS1 lasted 36 to 42 h, followed by a plateau period after 60 h, with a burst size of approximately 34 PFU per cell [29]. The latent period of MaMV-DC ranged from 24 to 48 h, with a burst size of about 80 infectious units per cell [23]. Ma-LBP had a latent period of 11.2 h, yielding an average burst size of 28 viral particles per host cell [26]. The latent period and burst size of Ma-LMM01 were estimated to range from 6 to 12 h and 50 to 120 infectious units per cell, respectively [34]. Me-ZS1 exhibited a latent period of 108 h, followed by a burst period of 46 h and a plateau period [28]. PhiMa05 had a latent period of 1 day, followed by a prolonged plateau period of approximately 3 days, with a burst size of 127 phage particles per infected cell; total lysis of the host required 4 days [27]; Mae-Yong1326-1 had a latent period of 3 h and a burst period of 33 h, with a burst size of 329 PFU per cell [20]. Compared to Microcystis cyanophages, Mae-Yong1326-1 had a shorter latent period and a larger burst size. Surprisingly, Mwe-Yong1112-1, YongM, and Mae-Yong1326-1 not only exhibited a broad spectrum of activity but also demonstrated high efficiency. This is of great significance for the management of algal blooms.

It is important to emphasize that if we aim to utilize broad-spectrum cyanophages for effective bloom management, prioritizing the stability of these cyanophages is essential. The stability of cyanophages plays a crucial role in maintaining their effectiveness in eliminating algae, even when there are fluctuations in temperature and pH levels in the water body. Studies have shown that most of the cyanophages mentioned above exhibit a high tolerance to temperature and pH, maintaining their activity within the range of 0–45 °C and pH 3–10 [29]. However, they are more sensitive to ultraviolet irradiation and organic solvents. This sensitivity may be attributed to the fact that ultraviolet rays can damage genetic material, while organic solvents can disrupt the protein shell [29]. Another key factor for using cyanophage in controlling cyanoHABs is the host growth stage. The growth conditions of the host significantly influence the reproduction of cyanophages. Cyanobacteria cultivated under eutrophic conditions exhibit elevated levels of global regulatory factors in the host, resulting in a higher concentration of protein nitrogen, which enhances the cyanophage’s ability to exploit and disrupt the host [3]. The number of virions added to the number of host cells is referred to as the multiplicity of infection (MOI), which plays a critical role. A relatively low MOI requirement (<1) appears to be an ideal condition for controlling blooms [3].

Frustratingly, the number of cyanophages reported so far is far lower than the demand for bloom management, regardless of whether they are high efficiency, broad spectrum, or both, which hinders the use of cyanophages for bloom management. Therefore, it is urgent to discover more efficient and broad-spectrum cyanophages, conduct genome annotation, predict infection modules, analyze the mechanism of action of key genes in infection modules, and elucidate the infection process and mechanism. This will facilitate the artificial transformation and utilization of cyanophages. 

## 3. Engineering Artificial Cyanophage and Gene Function Modules Mining

**Figure 2:**
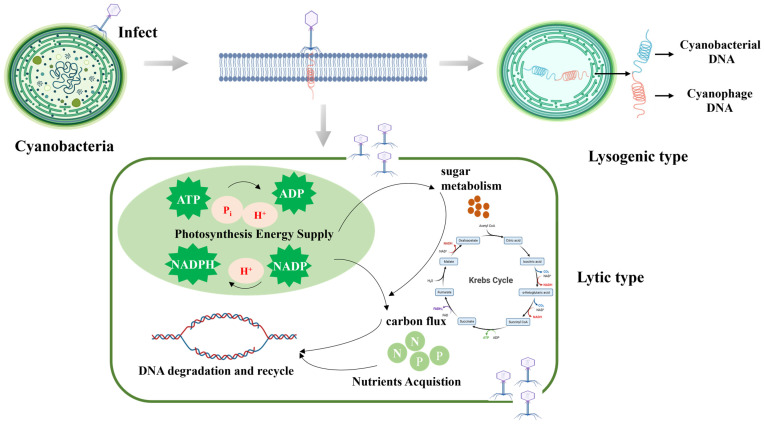
Cyanophage–cyanobacteria infection process and removal mechanism.

These phages utilize two main infection strategies known as lytic and lysogenic, and their infection mechanisms have been elucidated (Figure 2). When phages infect cyanobacteria, they inject their nucleic acid into the host cell. The injected nucleic acid then utilizes the resources of the host to replicate and assemble capsid proteins, leading to alterations in various biological processes within the host. These changes involve photosynthesis, sugar metabolism, carbon metabolism, DNA synthesis and repair, and nutrient acquisition.

There are few reports of attempted modification, artificial assembly of cyanophages, and the design of artificial cyanophages [36,37,38]. Firstly, only a few cyanophage genomes have been artificially assembled. For example, a truncated cyanophage Syn-P4-8 was assembled [38], as well as Syn-A-4-8 [36]. Additionally, the full-length cyanophage A-4L was de novo synthesized [37]. Currently, the genomes of phagocytic organisms are being streamlined, with mutants A-1(L) and A-4(L) engineered using the CRISPR-Cas12a system. In comparison to the wild type, these mutant genomes exhibit a reduction of 6.6%, corresponding to a deletion of 2778 bp [39]. Before this, no synthetic cyanophage genome has been constructed. Interestingly, there is a study indicating that compared with the control strain, the integration of a full-length cyanophage PP genome decreased photosynthesis and carbon fixation in model cyanobacterium *Synechococcus elongatus* PCC 7942, exhibiting cyanophage-like behavior [40]. But apart from this, there is no research into the infection mechanism of artificial cyanophages. Furthermore, since the synthesis of the first artificial *Mycoplasma mycoides* JCVI-syn1.0 [41], research into phages has been rapidly advancing. This includes phage therapy [42], phage-assisted continuous evolution [43,44], phage genome reduction [45], and more. It is worth noting that Zhou et al. [46,47] isolated a freshwater *Myoviridae* cyanophage Pam3 from Lake Chaohu, analyzed the structure and assembly of the tail fiber protein by cryo-electron microscopy, established a minimal phage, and in vitro revealed a redox-dependent mechanism of baseplate assembly and tail sheath contraction. It is a promising method to construct an artificial minimum cyanophage and use the concept of synthetic biology for engineering control.

By analyzing the function of gene modules in cyanophages, we can gain a deeper understanding of the essential genes required for successful infection and replication. This information can provide a valuable reference for the targeted deletion of redundant genes. Even though many cyanophage genomes have been annotated and resolved, there are still numerous hypothetical proteins in the cyanophage genome with unidentified functions. Guo et al. [48] conducted a rational analysis of the essential genes associated with the efficient broad-spectrum cyanophage YongM and successfully constructed its minimal genome. Although this minimal cyanophage is incapable of infecting host cyanobacteria, two critical genes, ORF1 thymidylate kinase and ORF50 primase, were identified during this investigation. Both enzymes play crucial roles in DNA replication, providing valuable insights for the development of a viable artificial cyanophage. Furthermore, through continuous UV mutagenesis of YongM, a variant with enhanced efficiency was obtained. Subsequent sequencing and analysis of the mutation site revealed that the phage-related tail fiber protein ORF83 is a key protein [48]. Chen et al. [38] enhanced the 5% NaCl tolerance of *Synechocystis* PCC6803 by introducing the truncated artificial cyanophage Syn-P4-8. Two key proteins, tail protein and tail fiber protein, were identified through transcriptome analysis. Meng et al. [49] demonstrated that the co-expression of two ORFs from cyanophage PaV-LD, ORF123 (encoding an endopeptidase) and ORF124 (encoding a membrane-associated protein, holin), inhibited the growth of *Synechocystis* PCC6803 and demonstrated the bacteriolytic effect of ORF123 and ORF124, contributing significantly to elucidating the bacteriolytic mechanism of cyanophages. Xiong et al. [50] proved that the cyanophage A-1(L) essential protein is crucial for the adsorption and infection of cyanophages to their hosts, *Anabaena* PCC 7120. The tail protein lipopolysaccharide-interacting protein (ORF36) specifically binds to the O antigen first, followed by ORF35 irreversibly binding to different sites. Nadel et al. [51] identified a crucial regulator called *non-bleaching A* (*nblA*) in a new marine cyanophage. NblA recruits proteases to phycobilisomes (PBS), leading to the replenishment of cells with nitrogen or sulfur. Interestingly, freshwater phage Ma-LMM01 contains a homolog of the *non-bleaching A* (*nblA*) gene [52]. 

In particular, through genomics analysis and the examination of genomic big data, a model of the interactions between cyanophages and cyanobacteria was developed. This model significantly enhances our understanding of the mechanisms underlying the broad-spectrum and efficient infectivity of cyanophages. Additionally, it facilitates the identification of key proteins involved in the infection process, thereby providing theoretical guidance for their practical applications. Zhang et al. [31] characterized the protein changes and regulatory networks during the infection. They found that metabolic pathways such as photosynthesis, precursor carbon, energy, and nitrogen supply were significantly altered. Key proteins involved included photosystem I P700 chlorophyll–apolipoprotein, carbon dioxide concentration mechanism protein, cytochrome B, and some infection lysis-related enzymes. Cyanophages contain auxiliary metabolic genes (AMGs), which enhance viral replication and dissemination by improving host metabolic pathways [53], including photosynthetic genes such as *psbA*, *psbD*, *cpeT*, and *hliP* [54,55,56]; electron carrier genes like *petE* [57], *pebS* [58], *ho1*, *pebA*, *petF*, and *pcyA* [59]; aldolase family genes that facilitate carbon flux, such as *talc* [54]; integrase gene *int* [54]; nucleotide synthesis genes *purB*, *purC*, *purH*, *purM*, *purN*, *purS, pyrE*, and *thyX* [55]; and phosphate-inducible genes *phoH* and *pstS* [54], as shown in Figure 2. Moreover, Lin et al. [60] constructed the Novel Cyanophage Genome Sequence Collection (NCGC), which facilitated a large-scale and comprehensive analysis of sequencing data from cyanobacteria and cyanophages. They discovered that the interactions between cyanobacteria and cyanophages in freshwater and marine ecosystems are interconnected. This significantly expands the repository of genetic information on cyanophages, enhancing our understanding of how environmental factors influence cyanobacteria–cyanophage interactions. Ultimately, this knowledge will guide us in effectively utilizing this information to manage algal blooms.

Interestingly, Shitrit et al. [61] reported a new method named REEP for recombination, enrichment, and PCR screening in engineering cyanophages. Using REEP, which relies on naturally occurring homologous recombination between conjugative plasmid and phage DNA during infection, it was found that the integrase and attachment site are necessary for integration into the host genome during the lysogenic life cycle. Furthermore, the increasing availability of gene editing tools for model cyanobacteria is essential for studying the interaction between cyanophages and cyanobacteria [62,63].

## 4. Application Status

Bacteriophage sterilization research is now advancing rapidly. Engineers can greatly improve the modularity of phagemids to alleviate *Chlamydia* trachomatis infection and bacterial infections [64] without the release of harmful endotoxins and adverse side effects [65]. These findings demonstrate that modified phagemids are a promising option for treating bacteria. The mechanism of infection by different phages is similar. The study of engineering phages provides valuable insights for cyanophages to control cyanoHABs. Promoting the flocculation, death, and precipitation of cyanobacteria, lowering algal toxin levels in water, and protecting aquatic organisms are important aspects of engineering modifications of cyanophages [3,66].

Fortunately, several small-scale experiments have successfully demonstrated the feasibility of using cyanophages to control cyanobacterial blooms. During these experiments, the growth of cyanobacteria was inhibited following the addition of cyanophages. However, there were no significant differences between the infected and control groups in terms of nitrogen-fixing rates or nitrogenase reductase gene expression levels. These results indicate that cyanophages can significantly influence the population dynamics of cyanobacteria with minimal impacts on nitrogen fixation [67]. Under optimal conditions, *M. aeruginosa* isolated from Lake Baroon was co-cultured with the natural lake cyanophage Ma-LBP. The abundance of the host decreased by 95% within 6 days; however, the cultured host community recovered within 3 weeks. The host may develop resistance and immunity, or this phenomenon may be related to the ratio of cyanophage to host [26]. Currently, the laboratory has conducted aquatic microcosm experiments with cyanophages. A suitable amount of lake water is introduced into the pool, along with a significant number of cyanophages. In the microcosm experiment, Me-ZS1 demonstrated a notable effect on reducing the relative abundance of cyanobacteria, increasing the relative abundance of *Saprospiraceae*, and protecting brocade carp (*Carassius auratus*) in cyanobacterial bloom water [28]. Cyanophages possess the ability to eliminate cyanobacteria in lakes. However, based on the limited examples of practical applications described above, the concept of using cyanophages to directly control cyanobacteria in lakes appears to be fraught with uncertainty. This uncertainty arises from the ongoing cycle of global water resources and biomass, in which cyanophages, cyanobacteria, and other aquatic species maintain mutual balance. As previously mentioned, the recovery observed after 3 weeks may represent a new equilibrium among these organisms. Unfortunately, there are currently no studies involving actual water testing.

Cyanophages, as viruses that specifically target cyanobacteria, have the potential to offer a highly specific control strategy with minimal impact on the environment. Cyanophages provide various benefits, including safety, a broad spectrum, novelty, high efficiency, stability, and sensitivity. However, there are still many challenges in using cyanophages—first and foremost, how to prevent escape [68]. Additionally, the infection mechanism is not clear, and research into artificial engineering is not in-depth [3,66].

## 5. Discussion

Here, we examined the advancements in cyanophage engineering for cyanophage control, aiming to offer fresh perspectives on cyanophage management. Compared with traditional methods, cyanophages are currently the most promising approach for bloom management. Cyanophages offer numerous advantages in bloom management and have minimal impact on the ecological environment and the geochemical cycle. Compared with the cyanophages that have been isolated so far, it is urgent to isolate and purify more broad-spectrum cyanophages. This is crucial for the future management of blooms containing various cyanobacteria. In addition, if it is necessary to expand the use of cyanophages, a large number of experiments are needed to verify the quantity of cyanophages, time, and other crucial factors. It is worth noting that with the development of synthetic biology, there are still several challenges in the comprehensive transformation of cyanophages using engineering concepts to make them artificially controllable. These challenges include how to obtain minimal artificial cyanophages with large genomes, how to prevent phage escape, and how to ensure that artificial cyanophages have the ability to infect hosts. In conclusion, this paper reviews the research progress on utilizing and modifying cyanophages to control blooms. It is believed that cyanophages will play an important role in managing future blooms in the near future.

## Figures and Tables

**Table 1:** Thirteen freshwater *Microcystis* cyanophages.

Name	Morphology	Genome Length (kb)	Infection Period (h)	Burst Size (PFU/Cell)	Accession Number	Characteristic	Spectrum			Reference
ΦMHI42	*Podoviridae*	-	-	-	-	Broad spectrum	*Microcystis* and *Planktothrix*	The concentration of *M. aeruginosa* began to decline 400 h after infection; *P. agardhii* stopped growing completely 5 days after infestation	Fishing lake in Hayling Island, Hampshire, UK	[18]
MaMV-DC	*Myoviridae*	169.223	24–48	80	NC_029002	Broad spectrum	*M. aeruginosa* FACHB-524, *M. flos-aquae* TF09, *M. aeruginosa* TA09 and *M. wesenbergii* DW09		Lake Dianchi, Kunming, China	[23]
Ma-LMM01	*Myoviridae*	162.109	6–12	50–120	NC_008562	-			Lake Mikata, Fukui Prefecture, Japan	[24]
Mic1	*Siphoviridae*	92.627	-	-	MN013189	-			Lake Chaohu, HeFei, China	[25]
Ma-LBP	*Podoviridae*	-	11.2	28	-	-			Lake Baroon, Queensland, Australia	[26]
PhiMa05	*Myoviridae*	27.3876	24	127	MW495066	-			Songklanagarind Hospital wastewater, Songkhla Province, Thailand	[27]
vB_MweS-yong2	Unassigned	44.530	-	-	OM681334	-			Yuehu Park, Ningbo, China	[20]
Me-ZS1	*Siphoviridae*	49.665	108	-	MK069556	Broad spectrum	Twelve strains across taxonomic orders: *Chroococcales*, *Nostocales*, and *Oscillatoriales*		A pond at Ningbo University, Ningbo, China	[28]
MinS1	*Siphoviridae*	49.966	36–42	34	MZ923504	Broad spectrum	Nineteen out of thirty cyanobacteria strains tested, containing five orders: *Chroococcales*, *Nostocales*, *Oscillatoriales*, *Hormogonales*, and *Synechococcales*		Mayang Stream, Fujian, China	[29]
Mae-Yong924-1	Unassigned	40.325	-	-	MZ447863	Broad spectrum	Six diverse cyanobacteria strains across three orders: *Chroococcales*, *Nostocales*, and *Oscillatoriales*	the host is lysed after 7–10 days	Yangming Lake in the Meishan campus of Ningbo University, Ningbo, China	[30]
Mae-Yong1326-1	Unassigned	48.822	3	329	OP028995	Broad spectrum, efficient	Seven cyanobacteria strains across three orders: *Chroococcales*, *Nostocales*, and *Oscillatoriales*		Lake Taihu, Suzhou, China	[20]
YongM	*-*	65.4	-	-	MT426122	Broad spectrum, efficient	Eighteen cyanobacteria strains across four orders: *Chroococcales*, *Nostocales*, *Oscillatoriales*, and *Synechococcales*	The cyanobacteria solution turned yellow after 8 h	Lake Dianchi, Kunming, China	[31]
Mwe-Yong1112-1	Unassigned	39.679	-	-	MZ436628	Broad spectrum, efficient	Twenty-three cyanobacterial strains across four different orders: *Chroococcales*, *Nostocales*, *Oscillatoriales*, and *Synechococcales*	Seven cyanobacterial strains were lysed completely within 3 days	A stream in the community, Ningbo, China	[32]

## Data Availability

No data were used for the research described in the article.

## References

[1] Bhatt P., Engel B.A., Reuhs M., Simsek H. (2023). Cyanophage technology in removal of cyanobacteria mediated harmful algal blooms: A novel and eco-friendly method. Chemosphere.

[2] Aranda Y.N., Bhatt P., Ates N., Engel B.A., Simsek H. (2023). Cyanophage-cyanobacterial interactions for sustainable aquatic environment. Environ. Res..

[3] Grasso C.R., Pokrzywinski K.L., Waechter C., Rycroft T., Zhang Y., Aligata A., Kramer M., Lamsal A. (2022). A Review of Cyanophage-Host Relationships: Highlighting Cyanophages as a Potential Cyanobacteria Control Strategy. Toxins.

[4] Zhu X., Li Z., Tong Y., Chen L., Sun T., Zhang W. (2023). From natural to artificial cyanophages: Current progress and application prospects. Environ. Res..

[5] Volk A., Lee J. (2023). Cyanobacterial blooms: A player in the freshwater environmental resistome with public health relevance?. Environ. Res..

[6] Diez-Quijada L., Casas-Rodriguez A., Guzmán-Guillén R., Molina-Hernández V., Albaladejo R.G., Cameán A.M., Jos A. (2022). Immunomodulatory Effects of Pure Cylindrospermopsin in Rats Orally Exposed for 28 Days. Toxins.

[7] Martin R.M., Bereman M.S., Marsden K.C. (2022). The Cyanotoxin 2,4-DAB Reduces Viability and Causes Behavioral and Molecular Dysfunctions Associated with Neurodegeneration in Larval Zebrafish. Neurotox. Res..

[8] Diez-Quijada L., Benítez-González M.D.M., Puerto M., Jos A., Cameán A.M. (2021). Immunotoxic Effects Induced by Microcystins and Cylindrospermopsin: A Review. Toxins.

[9] Drobac D., Svirev Z., Tokodi N., Vidovi M., Pavlica T.J.G.P. (2011). Microcystins: Potential risk factors in carcinogenesis of primary liver cancer in Serbia. Geogr. Pannonica.

[10] Stroom J.M., Kardinaal W.E.A. (2016). How to combat cyanobacterial blooms: Strategy toward preventive lake restoration and reactive control measures. Aquat. Ecol..

[11] Matthijs H.C.P., Jančula D., Visser P.M., Maršálek B. (2016). Existing and emerging cyanocidal compounds: New perspectives for cyanobacterial bloom mitigation. Aquat. Ecol..

[12] Deng Y., Wu M., Zhang H., Zheng L., Acosta Y., Hsu T.-T.D. (2017). Addressing harmful algal blooms (HABs) impacts with ferrate(VI): Simultaneous removal of algal cells and toxins for drinking water treatment. Chemosphere.

[13] Visser P.M., Ibelings B.W., Bormans M., Huisman J. (2016). Artificial mixing to control cyanobacterial blooms: A review. Aquat. Ecol..

[14] Park J., Church J., Son Y., Kim K.-T., Lee W.H. (2017). Recent advances in ultrasonic treatment: Challenges and field applications for controlling harmful algal blooms (HABs). Ultrason. Sonochem..

[15] Tang Y.Z., Gobler C.J. (2011). The green macroalga, Ulva lactuca, inhibits the growth of seven common harmful algal bloom species via allelopathy. Harmful Algae.

[16] Vafa E., Tayebi L., Abbasi M., Azizli M.J., Bazargan-Lari R., Talaiekhozani A., Zareshahrabadi Z., Vaez A., Amani A.M., Kamyab H. (2023). A better roadmap for designing novel bioactive glasses: Effective approaches for the development of innovative revolutionary bioglasses for future biomedical applications. Environ. Sci. Pollut. Res..

[17] Shaalan H., Cattan-Tsaushu E., Li K., Avrani S. (2023). Sequencing the genomes of LPP-1, the first isolated cyanophage, and its relative LPP-2 reveal different integration mechanisms in closely related phages. Harmful Algae.

[18] Watkins S.C., Smith J.R., Hayes P.K., Watts J.E. (2014). Characterisation of host growth after infection with a broad-range freshwater cyanopodophage. PLoS ONE.

[19] Harke M.J., Steffen M.M., Gobler C.J., Otten T.G., Wilhelm S.W., Wood S.A., Paerl H.W. (2016). A review of the global ecology, genomics, and biogeography of the toxic cyanobacterium, *Microcystis* spp.. Harmful Algae.

[20] Wang F., Li D., Cai R., Pan L., Zhou Q., Liu W., Qian M., Tong Y. (2022). A Novel Freshwater Cyanophage Mae-Yong1326-1 Infecting Bloom-Forming Cyanobacterium *Microcystis aeruginosa*. Viruses.

[21] Lee T.A., Rollwagen-Bollens G., Bollens S.M., Faber-Hammond J.J. (2015). Environmental influence on cyanobacteria abundance and microcystin toxin production in a shallow temperate lake. Ecotoxicol. Environ. Saf..

[22] Zhu J., Yang F., Du K., Wei Z.L., Wu Q.F., Chen Y., Li W.F., Li Q., Zhou C.Z. (2023). Phylogenomics of five *Pseudanabaena cyanophages* and evolutionary traces of horizontal gene transfer. Environ. Microbiome.

[23] Ou T., Li S., Liao X., Zhang Q. (2013). Cultivation and characterization of the MaMV-DC cyanophage that infects bloom-forming cyanobacterium *Microcystis aeruginosa*. Virol. Sin..

[24] Yoshida T., Nagasaki K., Takashima Y., Shirai Y., Tomaru Y., Takao Y., Sakamoto S., Hiroishi S., Ogata H. (2008). Ma-LMM01 infecting toxic *Microcystis aeruginosa* illuminates diverse cyanophage genome strategies. J. Bacteriol..

[25] Yang F., Jin H., Wang X.Q., Li Q., Zhang J.T., Cui N., Jiang Y.L., Chen Y., Wu Q.F., Zhou C.Z. (2020). Genomic Analysis of Mic1 Reveals a Novel Freshwater Long-Tailed Cyanophage. Front. Microbiol..

[26] Tucker S., Pollard P. (2005). Identification of Cyanophage Ma-LBP and Infection of the Cyanobacterium *Microcystis aeruginosa* from an Australian Subtropical Lake by the Virus. Appl. Environ. Microbiol..

[27] Naknaen A., Suttinun O., Surachat K., Khan E., Pomwised R. (2021). A Novel Jumbo Phage PhiMa05 Inhibits Harmful Microcystis sp.. Front. Microbiol..

[28] Lin W., Li D., Sun Z., Tong Y., Yan X., Wang C., Zhang X., Pei G. (2020). A novel freshwater cyanophage vB_MelS-Me-ZS1 infecting bloom-forming cyanobacterium *Microcystis elabens*. Mol. Biol. Rep..

[29] Zhang S., He X., Cao L., Tong Y., Zhao B., An W. (2022). A Novel Wide-Range Freshwater Cyanophage MinS1 Infecting the Harmful Cyanobacterium Microcystis aeruginosa. Viruses.

[30] Qian M., Li D., Lin W., Pan L., Liu W., Zhou Q., Cai R., Wang F., Zhu J., Tong Y. (2022). A Novel Freshwater Cyanophage, Mae-Yong924-1, Reveals a New Family. Viruses.

[31] Zhang S., Zhao B., Li J., Song X., Tong Y., An W. (2022). Host Cyanobacteria Killing by Novel Lytic Cyanophage YongM: A Protein Profiling Analysis. Microorganisms.

[32] Cai R., Li D., Lin W., Qin W., Pan L., Wang F., Qian M., Liu W., Zhou Q., Zhou C. (2022). Genome sequence of the novel freshwater *Microcystis cyanophage* Mwe-Yong1112-1. Arch. Virol..

[33] Wang J., Bai P., Li Q., Lin Y., Huo D., Ke F., Zhang Q., Li T., Zhao J. (2019). Interaction between cyanophage MaMV-DC and eight Microcystis strains, revealed by genetic defense systems. Harmful Algae.

[34] Yoshida T., Takashima Y., Tomaru Y., Shirai Y., Takao Y., Hiroishi S., Nagasaki K. (2006). Isolation and characterization of a cyanophage infecting the toxic cyanobacterium *Microcystis aeruginosa*. Appl. Environ. Microbiol..

[35] Zhou Q., Li D., Lin W., Pan L., Qian M., Wang F., Cai R., Qu C., Tong Y. (2023). Genomic Analysis of a New Freshwater Cyanophage Lbo240-yong1 Suggests a New Taxonomic Family of Bacteriophages. Viruses.

[36] Liu S., Feng J., Sun T., Xu B., Zhang J., Li G., Zhou J., Jiang J. (2022). The Synthesis and Assembly of a Truncated Cyanophage Genome and Its Expression in a Heterogenous Host. Life.

[37] Zhang T., Xu B., Feng J., Ge P., Li G., Zhang J., Zhou J., Jiang J. (2023). Synthesis and assembly of full-length cyanophage A-4L genome. Synth. Syst. Biotechnol..

[38] Chen Y., Ge P., Sun T., Feng J., Li G., Zhang J., Zhou J., Jiang J. (2023). Coexpression of Tail Fiber and Tail Protein Genes of the Cyanophage PP Using a Synthetic Genomics Approach Enhances the Salt Tolerance of Synechocystis PCC 6803. Microbiol. Spectr..

[39] Yuan S., Li Y., Kou C., Sun Y., Ma Y. (2024). CRISPR/Cas-based genome editing for cyanophage of Anabeana sp.. bioRxiv.

[40] Li G., Feng J., Zhu X., Chai Y., Sun T., Jiang J. (2024). Expression and characterization of the complete cyanophage genome PP in the heterologous host *Synechococcus elongatus* PCC 7942. bioRxiv.

[41] Hutchison C.A., Chuang R.Y., Noskov V.N., Assad-Garcia N., Deerinck T.J., Ellisman M.H., Gill J., Kannan K., Karas B.J., Ma L. (2016). Design and synthesis of a minimal bacterial genome. Science.

[42] Klumpp J., Dunne M., Loessner M.J. (2023). A perfect fit: Bacteriophage receptor-binding proteins for diagnostic and therapeutic applications. Curr. Opin. Microbiol..

[43] Esvelt K.M., Carlson J.C., Liu D.R. (2011). A system for the continuous directed evolution of biomolecules. Nature.

[44] Wei T., Lai W., Chen Q., Zhang Y., Sun C., He X., Zhao G., Fu X., Liu C. (2022). Exploiting spatial dimensions to enable parallelized continuous directed evolution. Mol. Syst. Biol..

[45] Yuan S., Shi J., Jiang J., Ma Y. (2022). Genome-scale top-down strategy to generate viable genome-reduced phages. Nucleic Acids Res..

[46] Yang F., Jiang Y.L., Zhang J.T., Zhu J., Du K., Yu R.C., Wei Z.L., Kong W.W., Cui N., Li W.F. (2023). Fine structure and assembly pattern of a minimal myophage Pam3. Proc. Natl. Acad. Sci. USA.

[47] Wei Z.L., Yang F., Li B., Hou P., Kong W.W., Wang J., Chen Y., Jiang Y.L., Zhou C.Z. (2022). Structural Insights into the Chaperone-Assisted Assembly of a Simplified Tail Fiber of the Myocyanophage Pam3. Viruses.

[48] Guo Y., Dong X., Li H., Lin W., Cao L., Li D., Zhang Y., Jin J., Tong Y., Liu Z. (2024). Efficient Broad-Spectrum Cyanophage Function Module Mining. Microorganisms.

[49] Meng L.H., Ke F., Zhang Q.Y., Zhao Z. (2022). Functional Analysis of the Endopeptidase and Holin from Planktothrix agardhii Cyanophage PaV-LD. Front. Microbiol..

[50] Xiong Z., Wang Y., Dong Y., Zhang Q., Xu X. (2019). Cyanophage A-1(L) Adsorbs to Lipopolysaccharides of Anabaena sp. Strain PCC 7120 via the Tail Protein Lipopolysaccharide-Interacting Protein (ORF36). J. Bacteriol..

[51] Nadel O., Rozenberg A., Flores-Uribe J., Larom S., Schwarz R., Béjà O. (2019). An uncultured marine cyanophage encodes an active phycobilisome proteolysis adaptor protein NblA. Environ. Microbiol. Rep..

[52] Meza-Padilla I., McConkey B.J., Nissimov J.I. (2024). Structural models predict a significantly higher binding affinity between the NblA protein of cyanophage Ma-LMM01 and the phycocyanin of Microcystis aeruginosa NIES-298 compared to the host homolog. Virus Evol..

[53] Ain Q.u., Wu K., Wu X., Bai Q., Li Q., Zhou C.-Z., Wu Q. (2024). Cyanophage-encoded auxiliary metabolic genes in modulating cyanobacterial metabolism and algal bloom dynamics. Front. Virol..

[54] Sullivan M.B., Coleman M.L., Weigele P., Rohwer F., Chisholm S.W. (2005). Three Prochlorococcus cyanophage genomes: Signature features and ecological interpretations. PLoS Biol..

[55] Fuchsman C.A., Carlson M.C.G., Garcia Prieto D., Hays M.D., Rocap G. (2021). Cyanophage host-derived genes reflect contrasting selective pressures with depth in the oxic and anoxic water column of the Eastern Tropical North Pacific. Environ. Microbiol..

[56] Ślesak I., Ślesak H. (2024). From cyanobacteria and cyanophages to chloroplasts: The fate of the genomes of oxyphototrophs and the genes encoding photosystem II proteins. New Phytol..

[57] Puxty R.J., Millard A.D., Evans D.J., Scanlan D.J. (2015). Shedding new light on viral photosynthesis. Photosynth. Res..

[58] Busch A.W., Reijerse E.J., Lubitz W., Hofmann E., Frankenberg-Dinkel N. (2011). Radical mechanism of cyanophage phycoerythrobilin synthase (PebS). Biochem. J..

[59] Dammeyer T., Bagby S.C., Sullivan M.B., Chisholm S.W., Frankenberg-Dinkel N. (2008). Efficient Phage-Mediated Pigment Biosynthesis in Oceanic Cyanobacteria. Curr. Biol..

[60] Lin W., Li D., Pan L., Li M., Tong Y. (2024). Cyanobacteria-cyanophage interactions between freshwater and marine ecosystems based on large-scale cyanophage genomic analysis. Sci. Total Environ..

[61] Shitrit D., Hackl T., Laurenceau R., Raho N., Carlson M.C.G., Sabehi G., Schwartz D.A., Chisholm S.W., Lindell D. (2022). Genetic engineering of marine cyanophages reveals integration but not lysogeny in T7-like cyanophages. ISME J..

[62] Gao X., Sun T., Pei G., Chen L., Zhang W. (2016). Cyanobacterial chassis engineering for enhancing production of biofuels and chemicals. Appl. Microbiol. Biotechnol..

[63] Sun T., Li S., Song X., Diao J., Chen L., Zhang W. (2018). Toolboxes for cyanobacteria: Recent advances and future direction. Biotechnol. Adv..

[64] Bhattarai S.R., Yoo S.Y., Lee S.-W., Dean D. (2012). Engineered phage-based therapeutic materials inhibit Chlamydia trachomatis intracellular infection. Biomaterials.

[65] Krom R.J., Bhargava P., Lobritz M.A., Collins J.J. (2015). Engineered Phagemids for Nonlytic, Targeted Antibacterial Therapies. Nano Lett..

[66] McKindles K.M., Manes M.A., DeMarco J.R., McClure A., McKay R.M., Davis T.W., Bullerjahn G.S. (2020). Dissolved Microcystin Release Coincident with Lysis of a Bloom Dominated by Microcystis spp. in Western Lake Erie Attributed to a Novel Cyanophage. Appl. Environ. Microbiol..

[67] Kuznecova J., Šulčius S., Vogts A., Voss M., Jürgens K., Šimoliūnas E. (2020). Nitrogen Flow in Diazotrophic Cyanobacterium Aphanizomenon flos-aquae Is Altered by Cyanophage Infection. Front. Microbiol..

[68] Zhou Y., Xu X., Wei Y., Cheng Y., Guo Y., Khudyakov I., Liu F., He P., Song Z., Li Z. (2021). A widespread pathway for substitution of adenine by diaminopurine in phage genomes. Science.

